# Genetic dissection of maize drought tolerance for trait improvement

**DOI:** 10.1007/s11032-020-01194-w

**Published:** 2021-01-19

**Authors:** Shengxue Liu, Feng Qin

**Affiliations:** College of Biological Sciences, China Agricultural University, Beijing, 100193 China

**Keywords:** QTL, GWAS, Drought tolerance, Molecular breeding, Drought stress, Maize

## Abstract

Maize is one of the most important crops, but its production is threatened by drought stress worldwide. Thus, increased drought tolerance has been a major goal of maize breeding. Conventional breeding strategies have led to significantly increase of maize yields; however, these strategies often fail to meet the need for drought stress tolerance enhancement. Here, we focus on progress related to the genetic dissection of drought tolerance in maize at different developmental stages achieved through linkage mapping and association mapping. Moreover, recent molecular breeding systems, including transgenic, genome-wide marker-assisted selection, and genome editing technologies, have provided a more direct, efficient, and accurate approach for trait improvement. We also provide perspectives on future directions regarding multi-omics studies and maize improvement. Overall, the application of acquired knowledge will facilitate maize breeding to meet the challenges.

## Introduction

Maize (*Zea mays* L.) is a primary cereal crop and is cultivated for food, feed, and industrial materials. Although maize yields have increased significantly in modern times, its production in all stages of plant growth, especially flowering and grain filling, is threatened globally by drought (Boyer and Westgate 2004; Lobell et al. 2014). Germination, emergence, seedling establishment, vegetative growth and development, and reproductive growth are all sensitive to drought stress. Thus, increased drought tolerance has been a major goal of maize breeding. Germination in maize varieties begins to markedly decline at − 0.99 MPa of solution osmotic potential, and this level of osmotic potential is also associated with poor post-germination performance (Liu et al. 2015). The effect of drought on seedling growth is intensively studied since subsequent development may be irreversibly impacted (Maiti et al. 1996). In spring and early summer in arid and semiarid regions such as northern China, crops often undergo drought stress when water deficits threaten germination and seedling growth. Therefore, it is essential to improve the drought tolerance of maize at the primary growth stages to improve establishment and subsequent growth. Moreover, when drought episode happens in reproductive growth, it typically results in asynchronous development of anthesis and silking (known as anthesis and silking interval, ASI) (Bruce et al. 2002; Tuberosa et al. 2002). The drought-induced ASI hampers successful pollination and greatly reduces the grain yields. Thus, there is tremendous interest in genetic dissection of ASI and correlated secondary traits, such as kernel number and hundred-kernel weight. In addition, an important feature of water stress is that the hyperosmotic signal causes the accumulation of the phytohormone abscisic acid (ABA), which in turn elicits adaptive responses in plants (Zhu 2016).

Although conventional breeding strategies have led to the release of many new maize varieties, these strategies often fail to meet the need for improved yield and stress tolerance (Tester and Langridge 2010). Over the past decade, however, significant new information has been gained on the alleles that contribute to these traits. Most recently, molecular breeding systems, including transgenic, genome-wide marker-assisted selection, and gene-editing technologies, have provided a more direct, efficient, and accurate approach for trait improvement.

In this review, we focus on recent progress related to the genetic dissection of drought tolerance in maize at different developmental stages achieved through linkage mapping and association mapping. In addition, this review also provides an overview of recent progress in drought improvement in maize using genetic engineering combined with conventional breeding strategies.

## The physiological and morphological adaptations of maize under water-stressed conditions

**Fig. 1:**
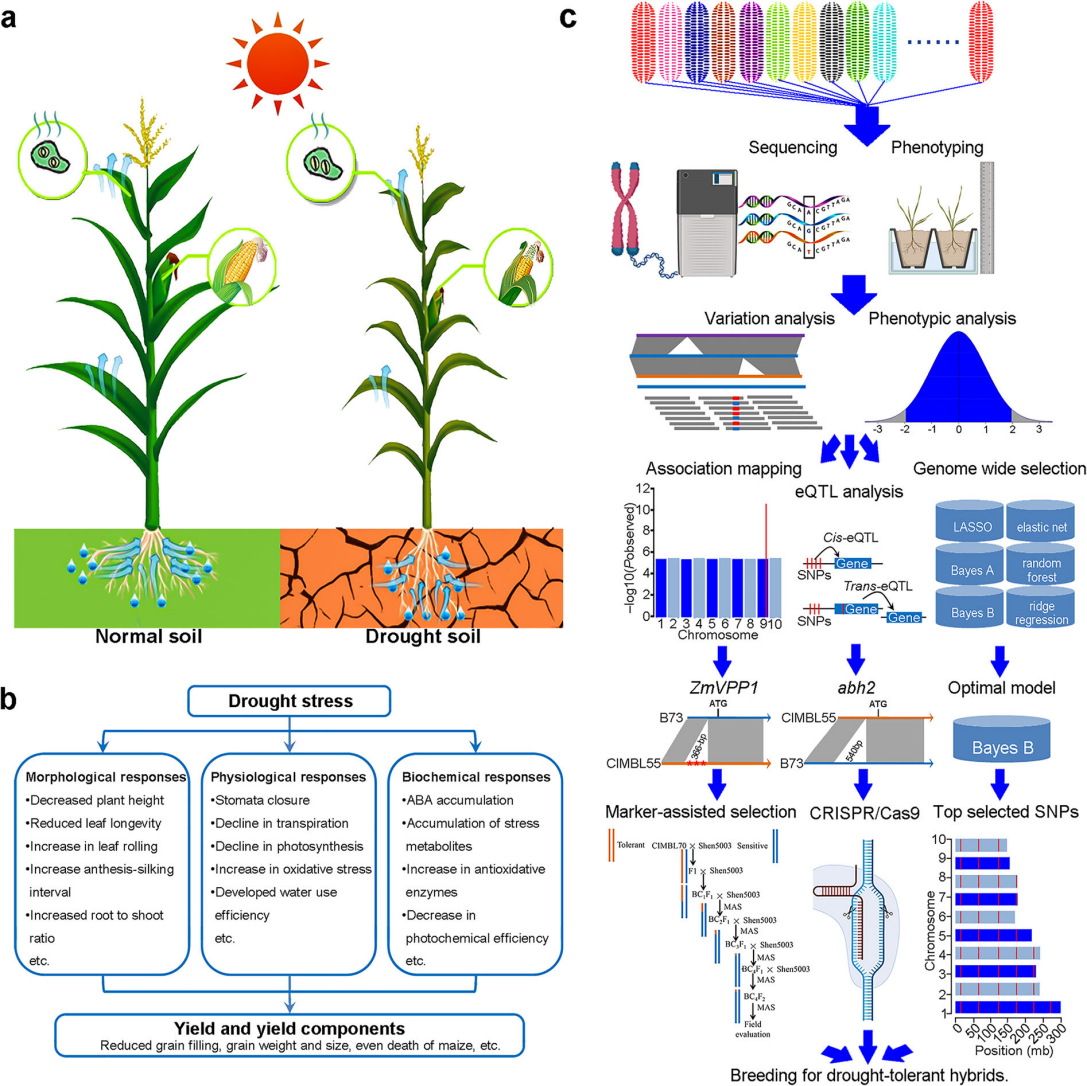
Effect of drought stress on maize growth and development and the research strategy for the trait improvement. **a** An illustration describing the morphological changes that occur in plants in response to drought stress. **b** The physiological and cellular responses that occur in maize in response water-deficit conditions and lead to reductions in growth and yield. **c** Schematic of the research strategy employed in genetic dissection of maize drought resistance for trait enhancement

Crops possess numerous physiological and molecular mechanisms to adapt to environmental stress that have been obtained through the course of natural and artificial selection (Fig. 1a). Responses to drought stress are dependent on plant species, the stage of plant development, the rate of dehydration, and the duration and severity of the drought stress. Drought resistance is generally defined as the ability of a plant to perceive a water deficiency and initiate coping strategies. It is a complex trait that encompasses three mechanisms: “drought escape” (completion of its life cycle prior to the detrimental effect of drought or undergo a period of dormancy until suitable conditions return); “drought avoidance” (the ability to maintain a relatively high tissue water content despite reduced soil water availability); and “drought tolerance” (ability to maintain cellular homeostasis through adaptive traits despite low water potential) (Levitt 1980). Leaf-rolling and survival rate are two common physiological indexes that are used to measure drought tolerance at the seedling stage. Beginning at leaf water potentials of − 1 MPa, leaf-rolling is observed and reaches a maximum around − 2 MPa (Baret et al. 2018). It helps plant to reduce water loss and avoid further stress injury. If the severity of drought continues to increase, the level of plant death will also increase. Survival rate refers to the ability of the plant to maintain viability while being subjected to drought stress and resume normal growth when sufficient water becomes available. The survival rate of maize across different genetic resources was investigated to range from 1.65 to 82.98%, when severe drought stress was applied to the seedlings, indicating a great genetic diversity in the maize germplasm (Wang et al. 2016). At a cellular level, drought signals promote stomatal closure to save water, stimulate the production of stress-protectant metabolites, upregulate the antioxidant system, and deploy peroxidase enzymes to prevent acute cellular damage and loss of membrane integrity (Gupta et al. 2020) (Fig. 1b).

Crop yield is vulnerable when drought conditions occur during the reproductive phase of plant growth. Although grain yield (GY) when plants subjected to water stress is the final target trait used to assess the degree of drought tolerance, correlated traits, such as ASI and kernel number per row (KNR), are considered to have a higher heritability and thus may be more suitable as target traits for improving maize drought resistance (Monneveux et al. 2008; Xue et al. 2013; Jia et al. 2020). A universal response in maize to drought stress is a delay in silking in relation to pollen shed, which is a critical index for drought tolerance in maize genotypes, and has been shown to be highly correlated with grain yield under water-stressed conditions (Bruce et al. 2002).

Roots are the essential organ for perceiving water deficit signals and water uptake. Maize seminal roots are initiated after germination, and a nodal root system is subsequently developed. The uptake of water and nutrients is initially carried out by the seminal root system. The first nodal roots that form aboveground will penetrate the soil to provide support and keep the plant upright (Zhang et al. 2018b). Plant species, such as maize, have two general mechanisms about nutrient acquisition: the distribution of the root system and the efficiency of transmembrane nutrient uptake. Larger root cortical cell size and fewer root cortical cell file number were characterized to beneficial for drought tolerance by reducing the metabolic cost of soil exploration and enabling deeper soil exploration (Chimungu et al. 2014a, b). Reduced crown root number and lateral root branching density can improve drought tolerance in maize by increasing rooting depth and water acquisition from the subsoil (Zhan et al. 2015; Gao and Lynch 2016).

## Genetic dissection of drought tolerance in maize

Linkage mapping and genome-wide association studies (GWAS) are the two major strategies used in plants to identify QTLs for complex traits. A major objective in genetic mapping is to identify the causative gene(s) responsible for the phenotypic variation. Since the release of the maize B73 reference genome, GWAS and linkage mapping analyses in maize have substantially increased, resulting in the dissection of many agriculturally important traits. However, to identify the causal gene or variant is still challenging. To address this problem, new approaches, including Mendelian randomization (MR) analysis and transcriptome-wide association studies (TWAS), have been developed to analyze expression-trait associations and prioritize candidate genes associated with a trait based on variations in gene expression (Gusev et al. 2016; Zhu et al. 2016). These approaches have been used to conduct more comprehensive studies and analyses of gene expression architectures in response to different environmental conditions.

### QTL mapping of drought tolerance in maize

QTL mapping is a major method used to dissect the genetic basis of complex traits, and also serves as the basis of marker-assisted selection (MAS). Populations adopted for linkage mapping are usually derived from a bi-parental cross with clear ancestry. QTL mapping allows a researcher to determine if a chromosomal fragment between two specific breakpoints is associated with a specific phenotype. Molecular markers, such as restriction fragment length polymorphisms (RFLPs), random amplified polymorphism DNA (RAPD), sequence-characterized amplified regions, and simple sequence repeats (SSRs), have been developed and used for QTL analysis beginning in the last century (Kim et al. 2000). Currently, single nucleotide polymorphisms (SNPs) have taken the place of DNA fragment markers, as they encompass the greatest level of variation present in the genomes of organisms (Rafalski 2002). Genotyping-by-sequencing (GBS) focuses on the sequencing data obtained from DNA restriction fragments rather than the whole genome and allows researchers and breeders to identify genomic variations among many individuals of organisms with large genomes (Gore et al. 2009). Several studies have conducted QTL mapping of drought resistance in maize (Table 1). Early seedling growth is very important for maize establishment and grain yield production. Identification of quantitative trait loci for drought tolerance at seedling stage will contribute to the profile of drought tolerance throughout the maize life cycle and help understand the complex mechanism of this important agronomic trait. Seven QTLs related to survival rate have been reported on chromosomes 3, 4, 6, 7, and 9 that were identified based on of 93 SSR markers using multiple parents introgression lines (Hao et al. 2009). Nine QTLs related to leaf temperature have been reported on chromosomes 1, 2, 9, and 10 that were identified based on of 248 SSR markers using 187 recombinant inbred lines (RILs) derived from across of Zong3 (drought sensitive) × 87-1 (drought tolerant) genotypes (Liu et al. 2011).

**Table 1:** List of QTLs related to drought resistance identified by linkage mapping

Trait category	Phenotype	Mapping population	Sample size	Marker	Locus/QTL	Candidate gene	References
Seedling stage	SR	Multiple parents introgression lines	417	93 SSR	7 QTLs (umc1351, etc.)	None	Hao et al. 2009
	LTD, DTI, SFW, SDW	Zong 3 × 87-1	187	248 SSR	9 QTLs (bnlg1556, etc.)	None	Liu et al. 2011
Components of grain yield	GY, ASI, EAR	SD34 × SD35	230	70 RFLPs	11 QTLs (umc76, etc.)	None	Agrama and Moussa 1996
	MFLW, FFLW, ASI	Ac7643S_5_ × Ac7729/TZSRWS_5_	234	142 RFLPs	57 QTLs (umc119, etc.)	None	Ribaut et al. 1996
	EL, EW, KWE, KN, etc.	B73 × H99	142	173 RFLPs	34 QTLs (m16, etc.)	None	Frova et al. 1999
	L-ABA concentration	Os420 × IABO78	80	106 SSR	17 QTLs (umc11, etc.)	None	Sanguineti et al. 1999
	GY, ASI,	Huangzao 4 × Ye 107	184	89 SSR	20 QTLs (umc1160, etc.)	None	Li et al. 2003
	EL, KR, WK, KWP	Zong 3 × 87-1	221	261 SSR	92 QTLs (bnlg1014, etc.)	None	Lu et al. 2006
	MFLW, ASI, GY, KNO, etc.	CML444 × SC-Malawi	236	79 RFLPs and 81 SSR	57 QTLs (umc128, etc.)	None	Messmer et al. 2009
	GY, ASI	18 bi-parental populations	3130	from 118 to 202 SNPs	68 mQTLs	5 to 926 candidate genes in each mQTL	Semagn et al. 2013
	GY, ASI	3 bi-parental populations	781	1, 536 SNPs	7 mQTLs	16 (*ZmMADS16*, etc.)	Almeida et al. 2013
Root traits	L_ax_, L_Lat_, ER_ax_, K_Lat_, etc.	Ac7643 × Ac7729/TZSRW	208	132 RFLPs	13 QTLs (bin 2.02, etc.)	None	Ruta et al. 2010
	PRL, SRL, CRL, SRN, etc.	DH1M × T877	204	56, 000 SNPs	364 QTLs (SNP20, etc.)	None	Li et al. 2018

Abbreviations: *SR* survival rate, *LTD* leaf temperature differences, *DTI* drought tolerance index, *SFW* total shoot fresh weight, *SDW* shoot dry weight, *GY* grain yield, *ASI* anthesis-silking interval, *EAR* number of ears per plant, *MFLW* the time of male flowering, *FFLW* female flowering, *EL* ear length, *EW* ear weight, *KWE* kernel weight per ear, *KN* kernel number per ear, *KNO* the number of kernels per square meter, *Lax* axile root length, *L*_Lat_ lateral root length, *ERax* elongation rates of axile roots, *K*_Lat_ rate constant of lateral root elongation, *PRL* the length of primary root, *SRL* seminal root, *CRL* crown root, *SRN* the number of seminal roots

Due to the complex genetic basis of drought tolerance and poor heritability of the crop yield trait, individual trait components, such as ASI and KNR, are more frequently identified and characterized due to their better heritability in replicated experiments (Monneveux et al. 2008; Xue et al. 2013). Five QTLs related to grain yield have been reported on chromosomes 1, 3, 5, 6, and 8, which explain 50% of the phenotypic variance (Agrama and Moussa 1996). Ribaut et al. reported on QTLs associated with flowering time and ASI in maize under well-watered conditions and two water-stressed regimes (Ribaut et al. 1996). Based on 142 RILs derived from across between B73 (drought tolerant) × H99 (drought sensitive) maize genotypes, genomic segments were identified to be responsible for the expression of drought tolerance in yield component traits under well-watered and water-stressed conditions. Half of the QTLs were consistently detected in the two water-stressed regimes (Frova et al. 1999). Sanguineti et al. identified 17 QTLs controlling bulk-leaf ABA concentration (L-ABA) using 80 F_4_ random families derived from across between Os420 (high L-ABA parent) and IABO78 (low L-ABA parent) genotypes (Sanguineti et al. 1999). Two QTLs for ear setting were detected on chromosomes 3 and 6, under well-watered conditions, explaining approximately 19.9% of the phenotypic variance (Li et al. 2003). Lu et al. analyzed the additive and epistatic QTLs associated with yield and yield components based on 261 SSRs using 221 RILs as the test material under both well-watered and water-stressed conditions. Results indicated that many QTLs varied and suggested that this was most likely due to different levels of genetic expression exhibited in the two different water-stressed treatment groups (Lu et al. 2006). By analyzing a RIL population derived from across between CML444 (drought tolerant) and SC-Malawi (drought sensitive), 81, 57, 51, and 34 QTLs were uncovered for six target traits (male flowering, ASI, grain yield, kernel number, 100-kernel fresh weight, and plant height) (Messmer et al. 2009). A meta-QTLs (mQTLs) analysis was performed for grain yield and ASI across 18 bi-parental maize populations evaluated under both managed water-stressed and well-watered environments. Sixty-eight mQTLs were identified and each of them averagely explained 6.5% phenotypic variation (Semagn et al. 2013). Almeida et al. evaluated three tropical bi-parental populations under well-watered and water-stressed regimes. Across the three populations and multiple environments, seven genomic regions for grain yield and one for ASI were identified, with six mQTL on chr.1, 4, 5, and 10 for grain yield being constitutively expressed under both well-watered and water-stressed conditions (Almeida et al. 2013).

Root architecture and development has been shown to be a key component of drought tolerance (Pace et al. 2015). Understanding root development and the molecular mechanisms that influence root architecture is thus important for increasing yield potential and yield stability under varying environmental conditions and soil profiles (Hodge et al. 2009). Thirteen QTLs related to six root traits have been reported on chromosomes 2, 3, 5, 6, and 7 that were identified based on 208 RILs derived from across between two inbred lines, Ac7643 (drought tolerant) and Ac7729/TZSRW (drought sensitive) (Ruta et al. 2010). Li et al. evaluated 13 root and shoot traits and genetic plasticity based on 56,000 SNPs using a population of 204 recombinant inbred lines derived from across between two inbred lines, DH1M (drought tolerant) and T877 (drought sensitive) using single-seed descent. A total of 48 QTLs were identified, including 15 QTLs that were associated with 9 traits with significant QTL-by-Environment interactions (Li et al. 2018). Although many QTLs have been detected via linkage mapping, few studies report on the fine mapping of QTLs that enable the identification of the precise genetic position and/or the cloning of candidate gene(s). This is because large secondary populations are generally required to achieve sufficient map resolution, which require a high level of resources and are time-consuming to establish (Dinka et al. 2007). The large amounts of repetitive sequences in the maize genome have also hindered progress in QTL fine mapping and cloning (Jiao et al. 2017).

### Association mapping of drought-related QTLs

Maize is an ideal crop for the application of GWAS, and significant progress has been made in the last decade. The degree of linkage disequilibrium (LD), however, is a major factor affecting the resolution achieved by association mapping. Three versions of the maize haplotype map have been published, and the number of SNP variants on each of them were 3.3 million for 27 lines (Gore et al. 2009), 55 million SNPs for 103 lines (Chia et al. 2012), and up to 83 million for 1218 lines (Bukowski et al. 2018), respectively. The genome-wide LD decay (*r*^2^ < 0.2) in maize is ~ 5.5 kb in maize hapmap2 (Chia et al. 2012). Based on the rapid decay of linkage disequilibrium (LD) in the maize genome determined with high-quality SNPs, the use of GWAS has increased our understanding of the genetic architecture of complex quantitative traits and facilitated the cloning of genes underlying complex traits (Wang and Qin 2017).

Drought resistance is a complex polygenic trait, and its impact on crop production depends on the degree and duration of the reduced precipitation and soil water gradients, as well as on plant species and the developmental stage of plants. Association mapping of drought tolerance in maize seedlings has been reported in several studies (Liu et al. 2013; Mao et al. 2015; Wang et al. 2016; Zhang et al. 2019) (Table 2). Liu et al. analyzed all of the functional dehydration-responsive element-binding (DREB) protein genes in maize and examined their associations with natural variation in drought tolerance among 368 maize varieties collected from tropical and temperate regions. A significant association between the natural variation in *ZmDREB2.7* and drought tolerance was detected and specifically located in the gene promoter region which most likely enable the early induction of the gene expression (Liu et al. 2013). Mao et al. identified a miniature inverted-repeat transposable element (MITE) inserted in the promoter of a *NAC* gene (*ZmNAC111*) that was significantly associated with natural variation in maize drought tolerance. The MITE insertion is correlated with lower *ZmNAC111* expression in maize and suppresses *ZmNAC111* expression via the RNA-directed DNA methylation and H3K9 dimethylation pathway when it is expressed in *Arabidopsis* (Mao et al. 2015). Wang et al. reported on a GWAS analysis of maize drought tolerance at the seedling stage and identified 83 genetic variants that were resolved to 42 candidate genes. The peak SNP (chr9.S_94178074) overlapped with the previously reported QTL9.3 (Semagn et al. 2013) and is directly located in the *ZmVPP1*, which encodes a vacuolar-type H^+^ pyrophosphatase (H^+^-PPase) (Wang et al. 2016).

**Table 2:** List of QTLs related to drought resistance identified by association mapping

Trait category	Phenotype	Mapping population	Sample size	Marker numbers (SNP)	Candidate gene	References
Seedling stage	SR	Inbred lines populations	368	525, 105	*ZmDREB2.7*	Liu et al. 2013
			368	556, 944	*ZmNAC111*	Mao et al. 2015
			367	556, 944	*ZmVPP1*	Wang et al. 2016
Components of grain yield	ASI	Recombinant inbred line populations and inbred lines populations	961	2, 052	65 (GRMZM2G164325, etc.)	Lu et al. 2010
	GY, DTA, DTS, ASI, etc.	Inbred lines populations	350	56, 110	33 (GRMZM2G125777, etc.)	Xue et al. 2013
	GY, ASI, EG, EL, etc.	Inbred lines populations	240	29, 619	108 (GRMZM2G418217, etc.)	Thirunavukkarasu et al. 2014
	GY, DTA, DTS, ASI, etc.	Inbred lines populations	346	60, 000	16 (GRMZM2G035688, etc.)	Farfan et al. 2015
Root traits	TNR, RDW, CVA, PRL, etc.	Inbred lines populations	384	681, 257	GRMZM2G153722	Pace et al. 2015
	RDW, RL, RSA, RV, etc.	Inbred lines populations	396	955, 690	98 (GRMZM2G148106, etc.)	Zaidi et al. 2016
	RHL	Inbred lines populations	367	556, 944	*ZmTIP1*	Zhang et al. 2019
	PRL, SRL, SRN.	Inbred lines populations	209	43, 252	7 (GRMZM2G136364, etc.)	Guo et al. 2020

Abbreviations: *SR* survival rate, *DTA* days to anthesis, *DTS* days to silking, *ASI* anthesis-silking interval, *GY* grain yield, *EG* ear girth, *EL* ear length, *TNR* total number of roots, *RDW* root dry weight, *CVA* convex root area, *PRL* primary root length, *RDW* root dry weight, *RL* root length, *RSA* root surface area, *RV* root volume, *RHL* root hair length, *SRL* seminal root length, *SRN* seminal root number

Crop yield becomes especially vulnerable when drought stress occurs during the reproductive phase of plant development. Correlated secondary traits, such as ASI, kernel number, and hundred-kernel weight, are generally easier to measure and show a higher heritability and thus may represent a more suitable target for improving maize response to water stress (Xue et al. 2013). ASI is commonly used as a selection criterion for drought-tolerant maize genotypes, as it has been shown to be highly correlated with grain yield under water-stressed conditions (Lu et al. 2010; Xue et al. 2013; Thirunavukkarasu et al. 2014; Farfan et al. 2015) (Table 2). Lu et al. utilized 2,052 SNPs to screen across three RIL populations and 305 diverse inbred lines, under both well-watered and water-stressed conditions. This study represents a powerful approach for detecting QTLs, relative to other studies, due to the utilization of increased population size and allele diversity, as well as balanced allele frequencies (Lu et al. 2010). Xue et al. reported a GWAS of drought tolerance in maize at the reproductive phase of development and identified 42 associated SNPs located in 33 genes. GRMZM2G125777 was strongly associated with ear relative position, hundred-kernel weight, and timing of male and female flowering (Xue et al. 2013). Thirunavukkarasu et al. analyzed 240 accessions of subtropical maize under water-stressed conditions using 29,619 SNPs and identified genetic loci and their association with functional mechanisms. Maize gene models revealed that the SNPs mapped for agronomic traits were associated with a number of functional traits, including stomatal closure, flowering, root development, detoxification, and reduced water potential (Thirunavukkarasu et al. 2014). Farfan et al. used a diversity panel consisting of 346 maize inbred lines originating from temperate, sub-tropical, and tropical areas that were test crossed to stiff-stalk line Tx714 to conduct irrigated and non-irrigated trials for yield, plant height, ear height, days to anthesis, days to silking, and other agronomic traits. Three variants significantly explained 5–10% of the phenotypic variation in grain yield under both well-watered and water-stressed conditions (Farfan et al. 2015).

GWAS is also employed to analyze the allelic diversity underlying root characteristics and identify superior alleles (Table 2). Three hundred eighty-four inbred lines from the Ames panel were genotyped with 681,257 SNPs, and 22 seedling root architecture traits were phenotyped (Pace et al. 2015). The GWAS study identified 268 significantly associated SNPs. GRMZM2G153722, located on chromosome 4, contains 9 of the 13 significant SNPs identified for two traits, root diameter, and surface area (Pace et al. 2015). Zaidi et al. reported a GWAS of maize drought tolerance at the reproductive stage and identified 50 and 67 SNPs significantly associated with root functional (transpiration efficiency, flowering period water use) and structural (rooting depth, root dry weight, root length, root volume, root surface area, and root length density) traits, respectively (Zaidi et al. 2016). Guo et al. evaluated seminal root length (SRL) within an association panel consisting of 209 diverse maize accessions under well-watered and water-stressed conditions. They identified 7 candidate genes associated with seminal root development by integrating RNA-seq and GWAS data (Guo et al. 2020). Notably, transgenic maize with enhanced *ZmVPP1* and *ZmTIP1*expression exhibit enhanced root biomass and root hair elongation, respectively (Wang et al. 2016; Zhang et al. 2019), which also suggests that a better-developed root system may contribute to maize drought resistance.

### Transcriptome analysis facilitates genetic dissection for drought tolerance

Regulation of gene expression is fundamental aspect of stress response and adaptation. Thus, a transcriptomic approach has been widely used in studies of drought stress response in maize (Thatcher et al. 2014; Thatcher et al. 2016; Danilevskaya et al. 2019). A total of 94 RNA-seq libraries from ear, tassel, and leaves of the B73 public inbred line of maize were constructed at four developmental stages under both well-watered and water-stressed conditions to assess the effect of drought stress on developmentally regulated gene splicing. Results revealed that alternative splicing is strongly associated with tissue type, developmental stage, and stress condition (Thatcher et al. 2016). More than 200 Illumina RNA-seq libraries were constructed to identify transcripts in two different genotypes of maize in a variety of tissues (Thatcher et al. 2014). Many new alternatively spliced transcripts had the potential to code for entirely different proteins, which revealed a great diversity of protein sequences in the maize proteome (Thatcher et al. 2014). Parallel RNA-seq profiling of leaves, ears, and tassels of the maize B73 inbred line at several developmental stages of plants growing under field conditions revealed tissue-specific differences in response to drought stress. Tassel growth was reduced to a lesser extent than ear growth in response to drought stress. Genes controlling DNA replication, cell cycle, and cell division were significantly downregulated in stressed ears, which was consistent with the inhibition of ear growth in response to drought (Danilevskaya et al. 2019). Moreover, it is revealed that genetic variation in the regulatory region of *ZmNAC111*, *ZmVPP1*, and *ZmTIP1* affects gene expression in a manner that is associated with natural variation in drought tolerance in seedling stage (Mao et al. 2015; Wang et al. 2016; Zhang et al. 2019). To uncover the associations between the gene expression alteration with drought tolerance, TWAS and MR analyses are developed (Gusev et al. 2016; Zhu et al. 2016). A total of 627 RNA-seq analyses of maize leaf samples at the vegetative 2–3 stage that were collected from 224 maize accessions under three different water regimes was conducted to identify the regulatory variants controlling gene expression in response to drought. A total of 73,573 eQTLs were detected, and 60% of them were resolved to a single candidate gene. Importantly, 97 genes were prioritized in relation to their association with drought tolerance due to their variation in expression, which was identified through the MR analysis (Liu et al. 2020). Importantly, the natural variation in expression level of *abh2* (encoding an Abscisic acid 8′-hydroxylase) was identified to contribute to the seedling drought tolerance.

## Molecular breeding of drought resistance in maize

Drought resistance is a particularly complex quantitative trait controlled by many loci, each contributing a small effect. Conventional plant breeding has achieved genetic improved crops by crossing superior plants with other genotypes and conducting selection over years of field trials for enhanced yield performance under drought stress among the descendants (Babu et al. 2003). This is a tedious and long empirical process. New strategies and technologies are needed to improve the selection efficiency (Fig. 1c). Molecular breeding, including MAS, transgenics, gene editing, and genome-wide selection, is considered a promising approach for crop breeding to meet the growing demand for stress tolerant, higher yielding crop varieties (Gao 2014; Voytas and Gao 2014). The rounds of backcrossing required and linkage drag could be significantly reduced by marker-assisted background selection. Marker-assisted backcross selection of BC_2_F_3_ families (Ac7643 × CML247) were crossed with two testers (CML254 and CML274) and evaluated under different water regimes. Mean grain yield of the test hybrids was consistently at least 50% higher than control hybrids under severe water-stressed conditions (Ribaut and Ragot 2007). The superior allele of *ZmVPP1* from drought-tolerant inbred lines (CIMBL70 and CIMBL91) was introgressed into a drought-sensitive inbred line (Shen5003) through four generations of successive backcrossing of F1 plants (Shen5003 × CIMBL70 and Shen5003 × CIMBL91). For each generation, the *ZmVPP1*-heterozygous plants were selected and backcrossed with Shen5003. The homozygous-tolerant plants, NIL*-ZmVPP1*^CIMBL70^and NIL*-ZmVPP1*^CIMBL91^, were more tolerant than the NIL*-ZmVPP1*^Shen5003^ siblings (Wang et al. 2016).

Transgenic maize with improved drought resistance has been developed. Transgenic maize with increased *ZmNF-YB2* expression has been shown to confer drought tolerance and maintained photosynthetic capacity with improvements in grain yield observed across several growing seasons in fields under water-stressed conditions (Nelson et al. 2007). Overexpressing the *ZmASR1* increased in dry leaf weight and total chlorophyll content and improved maize kernel yield under well-watered and water-limited conditions in the field (Virlouvet et al. 2011). Expressing a gene encoding a rice trehalose-6-phosphate phosphatase (TPP) in developing ears increased both kernel set and harvest index under non-stress or drought conditions (Nuccio et al. 2015). The constitutive expression of two members of a family of bacterial RNA chaperones, *Escherichia coli* CspA and *Bacillus subtilis* CspB, was shown to confer abiotic stress tolerance in maize by improving growth, chlorophyll content, photosynthetic rate, and kernel numbers (Castiglioni et al. 2008). However, the suitability of the transgene, dosage effect, level of tolerance, yield penalty, and socio-scientific acceptance of transgenics in food crops like maize are all determinants that need to be evaluated when considering transgenic plant development (Cattivelli et al. 2008). Importantly, gene editing technology, which involves the use of site-specific nucleases engineered to modify target genes at a desirable location in the genome, represents a major step towards breeding by design (Rinaldo and Ayliffe 2015; Zhang et al. 2018a). Shi et al. employed Clustered Regularly Interspaced Short Palindromic Repeats (CRISPR)-Cas9 technology to generate novel variants of *ARGOS8*. As a result of this strategy, *ARGOS8* variants exhibited increased grain yield by as much as five bushels per acre, relative to non-engineered, wild-type plants under flowering stress conditions and had no yield loss under well-watered conditions (Shi et al. 2017). Liu et al. obtained three independent homozygous lines (*i1*, *d2*, and *d35*) using CRISPR-Cas9 technology. The survival rate of all three mutants under drought stress was significantly higher than wild-type plants (Liu et al. 2020). Taking the advantage of precise-targeting, gene editing technology is more plausive than gene transferring technology which usually introduces several kilo-base foreign DNA fragment to the genome. However, this technology relies on the strong knowledge on the superior allele of the major gene contributing to the trait. Thus, it emphasizes the importance of the genetic dissection and gene cloning of drought tolerance.

With the advent of new genomic tools, genome wide selection has also emerged as an important approach for complex trait improvement (Zhao et al. 2012; Shikha et al. 2017). Zhao et al. evaluated grain yield of F_3_ populations in 788 individuals using 960 SNPs. The prediction accuracy across populations was higher for grain moisture (0.90) than for grain yield (0.58) using random regression best linear unbiased prediction in combination with five-fold cross-validation (Zhao et al. 2012). Shikha et al. evaluated the breeding value of 240 subtropical lines of maize phenotyped for drought resistance under different environmental conditions using 29,619 SNPs. A total of 77 SNPs were associated with 10 drought-responsive transcription factors based on the predicting accuracy of seven genomic selection models (Shikha et al. 2017). These studies indicate that the cumulative effects of multiple quantitative resistance loci could potentially be exploited to produce high levels of drought tolerance.

## Future prospects

Over the past several decades, researches have shed light on the complex genetic architectures and regulatory mechanisms involved in drought resistance in maize. Drought-related QTL analyses have identified genomic regions that can be used for the direct selection of specific alleles. Meanwhile, GWAS has been used to identify hundreds of genetic variants in maize that are associated with drought-related traits. However, the candidate genes through which the identified genetic variants exert their effects on traits are remained largely unknown. Therefore, it is important to integrate multi-omic studies into linkage and association mapping to bridge the knowledge gap. Metabolomics provides a comprehensive high-throughput quantification of a broad range of metabolites and has been valuable for both phenotyping and diagnostic analyses in maize (Fernie and Schauer 2009; Riedelsheimer et al. 2012; Wen et al. 2014; Deng et al. 2020). Metabolomic data are also valuable for tracking evolutionary history and extending genomic insights into interspecific differentiation in maize and rice (Deng et al. 2020). Recent proteomics analysis has provided a functional context for the interpretation of gene expression variation in modern maize breeding (Jiang et al. 2019). The joint use of omics-data, specific genetic designs, and relevant analytical methods will provide integrative information that increase our understanding of the balance between stress response and grain yield and quality. Understanding the molecular regulatory mechanisms of drought response will also provide a useful foundation for maize breeding. With advances in the research topics presented in this review, the complex regulatory network associated with drought stress response and the evolution of drought resistance in maize ecotypes can be the subject of further studies in the future. Undoubtedly, this acquired knowledge will help fine-tune the development of new maize varieties that can adapt to and withstand the challenges presented by water scarcity.

## Data Availability

Not applicable.

## References

[CR1] Agrama HAS, Moussa ME (1996). Mapping QTLs in breeding for drought tolerance in maize (*Zea mays* L .). Euphytica.

[CR2] Almeida GD, Makumbi D, Magorokosho C, Nair S, Borem A, Ribaut JM, Banziger M, Prasanna BM, Crossa J, Babu R (2013). QTL mapping in three tropical maize populations reveals a set of constitutive and adaptive genomic regions for drought tolerance. Theor Appl Genet.

[CR3] Babu RC, Nguyen BD, Chamarerk V, Shanmugasundaram P, Chezhian P, Jeyaprakash P, Ganesh SK, Palchamy A, Sadasivam S, Sarkarung S, Wade LJ, Nguyen HT (2003). Genetic analysis of drought resistance in rice by molecular markers. Crop Sci.

[CR4] Baret F, Madec S, Irfan K, Lopez J, Comar A, Hemmerle M, Dutartre D, Praud S, Tixier MH (2018). Leaf-rolling in maize crops: from leaf scoring to canopy-level measurements for phenotyping. J Exp Bot.

[CR5] Boyer JS, Westgate ME (2004). Grain yields with limited water. J Exp Bot.

[CR6] Bruce WB, Edmeades GO, Barker TC (2002). Molecular and physiological approaches to maize improvement for drought tolerance. J Exp Bot.

[CR7] Bukowski R, Guo X, Lu Y, Zou C, He B, Rong Z, Wang B, Xu D, Yang B, Xie C, Fan L, Gao S, Xu X, Zhang G, Li Y, Jiao Y, Doebley JF, Ross-Ibarra J, Lorant A, Buffalo V, Romay MC, Buckler ES, Ware D, Lai J, Sun Q, Xu Y (2018). Construction of the third-generation Zea mays haplotype map. Gigascience.

[CR8] Castiglioni P, Warner D, Bensen RJ, Anstrom DC, Harrison J, Stoecker M, Abad M, Kumar G, Salvador S, D'Ordine R, Navarro S, Back S, Fernandes M, Targolli J, Dasgupta S, Bonin C, Luethy MH, Heard JE (2008). Bacterial RNA chaperones confer abiotic stress tolerance in plants and improved grain yield in maize under water-limited conditions. Plant Physiol.

[CR9] Cattivelli L, Rizza F, Badeck F-W, Mazzucotelli E, Mastrangelo AM, Francia E, Mare C, Tondelli A, Stanca AM (2008). Drought tolerance improvement in crop plants: an integrated view from breeding to genomics. Field Crops Res.

[CR10] Chia JM, Song C, Bradbury PJ, Costich D, de Leon N, Doebley J, Elshire RJ, Gaut B, Geller L, Glaubitz JC, Gore M, Guill KE, Holland J, Hufford MB, Lai J, Li M, Liu X, Lu Y, McCombie R, Nelson R, Poland J, Prasanna BM, Pyhajarvi T, Rong T, Sekhon RS, Sun Q, Tenaillon MI, Tian F, Wang J, Xu X, Zhang Z, Kaeppler SM, Ross-Ibarra J, McMullen MD, Buckler ES, Zhang G, Xu Y, Ware D (2012). Maize HapMap2 identifies extant variation from a genome in flux. Nat Genet.

[CR11] Chimungu JG, Brown KM, Lynch JP (2014). Large root cortical cell size improves drought tolerance in maize. Plant Physiol.

[CR12] Chimungu JG, Brown KM, Lynch JP (2014). Reduced root cortical cell file number improves drought tolerance in maize. Plant Physiol.

[CR13] Danilevskaya ON, Yu G, Meng X, Xu J, Stephenson E, Estrada S, Chilakamarri S, Zastrow-Hayes G, Thatcher S (2019). Developmental and transcriptional responses of maize to drought stress under field conditions. Plant Direct.

[CR14] Deng M, Zhang X, Luo J, Liu H, Wen W, Luo H, Yan J, Xiao Y (2020). Metabolomics analysis reveals differences in evolution between maize and rice. Plant J.

[CR15] Dinka SJ, Campbell MA, Demers T, Raizada MN (2007). Predicting the size of the progeny mapping population required to positionally clone a gene. Genetics.

[CR16] Farfan ID, De La Fuente GN, Murray SC, Isakeit T, Huang PC, Warburton M, Williams P, Windham GL, Kolomiets M (2015). Genome wide association study for drought, aflatoxin resistance, and important agronomic traits of maize hybrids in the sub-tropics. PLoS One.

[CR17] Fernie AR, Schauer N (2009). Metabolomics-assisted breeding: a viable option for crop improvement?. Trends Genet.

[CR18] Frova C, Krajewski P, Fonzo N, Villa M, Sari-Gorla M (1999). Genetic analysis of drought tolerance in maize by molecular markers I. yield components. Theor Appl Genet.

[CR19] Gao C (2014). Genome editing in crops: from bench to field. Natl Sci Rev.

[CR20] Gao Y, Lynch JP (2016). Reduced crown root number improves water acquisition under water deficit stress in maize (*Zea mays* L.). J Exp Bot.

[CR21] Gore MA, Chia JM, Elshire RJ, Sun Q, Ersoz ES, Hurwitz BL, Peiffer JA, McMullen MD, Grills GS, Ross-Ibarra J, Ware DH, Buckler ES (2009). A first-generation haplotype map of maize. Science.

[CR22] Guo J, Li C, Zhang X, Li Y, Zhang D, Shi Y, Song Y, Yang D, Wang T (2020). Transcriptome and GWAS analyses reveal candidate gene for seminal root length of maize seedlings under drought stress. Plant Sci.

[CR23] Gupta A, Rico-Medina A, Cano-Delgado AI (2020). The physiology of plant responses to drought. Science.

[CR24] Gusev A, Ko A, Shi H, Bhatia G, Chung W, Penninx BW, Jansen R, de Geus EJ, Boomsma DI, Wright FA, Sullivan PF, Nikkola E, Alvarez M, Civelek M, Lusis AJ, Lehtimaki T, Raitoharju E, Kahonen M, Seppala I, Raitakari OT, Kuusisto J, Laakso M, Price AL, Pajukanta P, Pasaniuc B (2016). Integrative approaches for large-scale transcriptome-wide association studies. Nat Genet.

[CR25] Hao Z, Liu X, Li X, Xie C, Li M, Zhang D, Zhang S, Xu Y (2009). Identification of quantitative trait loci for drought tolerance at seedling stage by screening a large number of introgression lines in maize. Plant Breed.

[CR26] Hodge A, Berta G, Doussan C, Merchan F, Crespi M (2009). Plant root growth, architecture and function. Plant Soil.

[CR27] Jia H, Li M, Li W, Liu L, Jian Y, Yang Z, Shen X, Ning Q, Du Y, Zhao R, Jackson D, Yang X, Zhang Z (2020). A serine/threonine protein kinase encoding gene KERNEL NUMBER PER ROW6 regulates maize grain yield. Nat Commun.

[CR28] Jiang LG, Li B, Liu SX, Wang HW, Li CP, Song SH, Beatty M, Zastrow-Hayes G, Yang XH, Qin F, He Y (2019). Characterization of proteome variation during modern maize breeding. Mol Cell Proteomics.

[CR29] Jiao Y, Peluso P, Shi J, Liang T, Stitzer MC, Wang B, Campbell MS, Stein JC, Wei X, Chin CS, Guill K, Regulski M, Kumari S, Olson A, Gent J, Schneider KL, Wolfgruber TK, May MR, Springer NM, Antoniou E, McCombie WR, Presting GG, McMullen M, Ross-Ibarra J, Dawe RK, Hastie A, Rank DR, Ware D (2017). Improved maize reference genome with single-molecule technologies. Nature.

[CR30] Kim NS, Park NI, Kim SH, Kim ST, Han SS, Kang KY (2000). Isolation of TC/AG repeat microsatellite sequences for fingerprinting rice blast fungus and their possible horizontal transfer to plant species. Mol Cells.

[CR31] Levitt J (1980). Responses of plants to environmental stresses. Water, radiation, salt, and other stresses.

[CR32] Li X, Liu X, Li M, Zhang S (2003). Identification of quantitative trait loci for anthesis-silking interval and yield components under drought stress in maize. Acta Bot Sin.

[CR33] Li P, Zhang Y, Yin S, Zhu P, Pan T, Xu Y, Wang J, Hao D, Fang H, Xu C, Yang Z (2018). QTL-by-environment interaction in the response of maize root and shoot traits to different water regimes. Front Plant Sci.

[CR34] Liu Y, Subhash C, Yan J, Song C, Zhao J, Li J (2011). Maize leaf temperature responses to drought: thermal imaging and quantitative trait loci (QTL) mapping. Environ Exp Bot.

[CR35] Liu S, Wang X, Wang H, Xin H, Yang X, Yan J, Li J, Tran LS, Shinozaki K, Yamaguchi-Shinozaki K, Qin F (2013). Genome-wide analysis of *ZmDREB* genes and their association with natural variation in drought tolerance at seedling stage of *Zea mays* L. PLoS Genet.

[CR36] Liu M, Li M, Liu K, Sui N (2015) Effects of drought stress on seed germination and seedling growth of different maize varieties. J Agric Sci 7(5). 10.5539/jas.v7n5p231

[CR37] Liu S, Li C, Wang H, Wang S, Yang S, Liu X, Yan J, Li B, Beatty M, Zastrow-Hayes G, Song S, Qin F (2020). Mapping regulatory variants controlling gene expression in drought response and tolerance in maize. Genome Biol.

[CR38] Lobell DB, Roberts MJ, Schlenker W, Braun N, Little BB, Rejesus RM, Hammer GL (2014). Greater sensitivity to drought accompanies maize yield increase in the U.S. Midwest. Science.

[CR39] Lu G-H, Tang J-H, Yan J-B, Ma X-Q, Li J-S, Chen S-J, Ma J-C, Liu Z-X, L-Z E, Zhang Y-R, Dai J-R (2006). Quantitative trait loci mapping of maize yield and its components under different water treatments at flowering time. J Integr Plant Biol.

[CR40] Lu Y, Zhang S, Shah T, Xie C, Hao Z, Li X, Farkhari M, Ribaut JM, Cao M, Rong T, Xu Y (2010). Joint linkage-linkage disequilibrium mapping is a powerful approach to detecting quantitative trait loci underlying drought tolerance in maize. Proc Natl Acad Sci U S A.

[CR41] Maiti RK, Maiti LE, Maiti S, Maiti AM, Maiti M, Maiti H (1996). Genotypic variability in maize cultivars (*Zea mays* L.) for resistance to drought and salinity at the seedling stage. Plant Physiol.

[CR42] Mao H, Wang H, Liu S, Li Z, Yang X, Yan J, Li J, Tran LS, Qin F (2015). A transposable element in a *NAC* gene is associated with drought tolerance in maize seedlings. Nat Commun.

[CR43] Messmer R, Fracheboud Y, Banziger M, Vargas M, Stamp P, Ribaut JM (2009). Drought stress and tropical maize: QTL-by-environment interactions and stability of QTLs across environments for yield components and secondary traits. Theor Appl Genet.

[CR44] Monneveux P, Sanchez C, Tiessen A (2008). Future progress in drought tolerance in maize needs new secondary traits and cross combinations. J Agric Sci.

[CR45] Nelson DE, Repetti PP, Adams TR, Creelman RA, Wu J, Warner DC, Anstrom DC, Bensen RJ, Castiglioni PP, Donnarummo MG, Hinchey BS, Kumimoto RW, Maszle DR, Canales RD, Krolikowski KA, Dotson SB, Gutterson N, Ratcliffe OJ, Heard JE (2007). Plant nuclear factor Y (NF-Y) B subunits confer drought tolerance and lead to improved corn yields on water-limited acres. Proc Natl Acad Sci U S A.

[CR46] Nuccio ML, Wu J, Mowers R, Zhou HP, Meghji M, Primavesi LF, Paul MJ, Chen X, Gao Y, Haque E, Basu SS, Lagrimini LM (2015). Expression of trehalose-6-phosphate phosphatase in maize ears improves yield in well-watered and drought conditions. Nat Biotechnol.

[CR47] Pace J, Gardner C, Romay C, Ganapathysubramanian B, Lubberstedt T (2015). Genome-wide association analysis of seedling root development in maize (*Zea mays* L.). BMC Genomics.

[CR48] Rafalski A (2002). Applications of single nucleotide polymorphisms in crop genetics. Curr Opin Plant Biol.

[CR49] Ribaut JM, Ragot M (2007). Marker-assisted selection to improve drought adaptation in maize: the backcross approach, perspectives, limitations, and alternatives. J Exp Bot.

[CR50] Ribaut JM, Hoisington DA, Deutsch JA, Jiang C, Gonzalez-de-Leon D (1996). Identification of quantitative trait loci under drought conditions in tropical maize. 1. Flowering parameters and the anthesis-silking interval. Theor Appl Genet.

[CR51] Riedelsheimer C, Lisec J, Czedik-Eysenberg A, Sulpice R, Flis A, Grieder C, Altmann T, Stitt M, Willmitzer L, Melchinger AE (2012). Genome-wide association mapping of leaf metabolic profiles for dissecting complex traits in maize. Proc Natl Acad Sci U S A.

[CR52] Rinaldo AR, Ayliffe M (2015). Gene targeting and editing in crop plants: a new era of precision opportunities. Mol Breeding.

[CR53] Ruta N, Liedgens M, Fracheboud Y, Stamp P, Hund A (2010). QTLs for the elongation of axile and lateral roots of maize in response to low water potential. Theor Appl Genet.

[CR54] Sanguineti RT, Landi P, Salvi S, Maccaferri M, Casarini ES (1999). QTL analysis of drought-related traits and grain yield in relation to genetic variation for leaf abscisic acid concentration in field-grown maize. J Exp Bot.

[CR55] Semagn K, Beyene Y, Warburton ML, Tarekegne A, Mugo S, Meisel B, Sehabiague P, Prasanna BM (2013). Meta-analyses of QTL for grain yield and anthesis silking interval in 18 maize populations evaluated under water-stressed and well-watered environments. BMC Genomics.

[CR56] Shi J, Gao H, Wang H, Lafitte HR, Archibald RL, Yang M, Hakimi SM, Mo H, Habben JE (2017). *ARGOS8* variants generated by CRISPR-Cas9 improve maize grain yield under field drought stress conditions. Plant Biotechnol J.

[CR57] Shikha M, Kanika A, Rao AR, Mallikarjuna MG, Gupta HS, Nepolean T (2017). Genomic selection for drought tolerance using genome-wide SNPs in maize. Front Plant Sci.

[CR58] Tester M, Langridge P (2010) Breeding technologies to increase crop production in a changing world. Science 327(5967):818–822. 10.1126/science.1183700 10.1126/science.1183700 20150489

[CR59] Thatcher SR, Zhou W, Leonard A, Wang BB, Beatty M, Zastrow-Hayes G, Zhao X, Baumgarten A, Li B (2014). Genome-wide analysis of alternative splicing in Zea mays: landscape and genetic regulation. Plant Cell.

[CR60] Thatcher SR, Danilevskaya ON, Meng X, Beatty M, Zastrow-Hayes G, Harris C, Van Allen B, Habben J, Li B (2016). Genome-wide analysis of alternative splicing during development and drought stress in maize. Plant Physiol.

[CR61] Thirunavukkarasu N, Hossain F, Arora K, Sharma R, Shiriga K, Mittal S, Mohan S, Namratha PM, Dogga S, Rani TS, Katragadda S, Rathore A, Shah T, Mohapatra T, Gupta HS (2014). Functional mechanisms of drought tolerance in subtropical maize (Zea mays L.) identified using genome-wide association mapping. BMC Genomics.

[CR62] Tuberosa R, Salvi S, Sanguineti MC, Landi P, Maccaferri M, Conti S (2002). Mapping QTLs regulating morpho-physiological traits and yield: case studies, shortcomings and perspectives in drought-stressed maize. Ann Bot.

[CR63] Virlouvet L, Jacquemot MP, Gerentes D, Corti H, Bouton S, Gilard F, Valot B, Trouverie J, Tcherkez G, Falque M, Damerval C, Rogowsky P, Perez P, Noctor G, Zivy M, Coursol S (2011). The *ZmASR1* protein influences branched-chain amino acid biosynthesis and maintains kernel yield in maize under water-limited conditions. Plant Physiol.

[CR64] Voytas DF, Gao C (2014). Precision genome engineering and agriculture: opportunities and regulatory challenges. PLoS Biol.

[CR65] Wang H, Qin F (2017). Genome-wide association study reveals natural variations contributing to drought resistance in crops. Front Plant Sci.

[CR66] Wang X, Wang H, Liu S, Ferjani A, Li J, Yan J, Yang X, Qin F (2016). Genetic variation in *ZmVPP1* contributes to drought tolerance in maize seedlings. Nat Genet.

[CR67] Wen W, Li D, Li X, Gao Y, Li W, Li H, Liu J, Liu H, Chen W, Luo J, Yan J (2014). Metabolome-based genome-wide association study of maize kernel leads to novel biochemical insights. Nat Commun.

[CR68] Xue Y, Warburton ML, Sawkins M, Zhang X, Setter T, Xu Y, Grudloyma P, Gethi J, Ribaut JM, Li W, Zheng Y, Yan J (2013). Genome-wide association analysis for nine agronomic traits in maize under well-watered and water-stressed conditions. Theor Appl Genet.

[CR69] Zaidi PH, Seetharam K, Krishna G, Krishnamurthy L, Gajanan S, Babu R, Zerka M, Vinayan MT, Vivek BS (2016). Genomic regions associated with root traits under drought stress in tropical maize (*Zea mays* L.). PLoS One.

[CR70] Zhan A, Schneider H, Lynch JP (2015). Reduced lateral root branching density improves drought tolerance in maize. Plant Physiol.

[CR71] Zhang Y, Massel K, Godwin ID, Gao C (2018). Applications and potential of genome editing in crop improvement. Genome Biol.

[CR72] Zhang Z, Zhang X, Lin Z, Wang J, Xu M, Lai J, Yu J (2018). The genetic architecture of nodal root number in maize. Plant J.

[CR73] Zhang X, Mi Y, Mao H, Liu S, Chen L, Qin F (2019). Genetic variation in *ZmTIP1* contributes to root hair elongation and drought tolerance in maize. Plant Biotechnol J.

[CR74] Zhao Y, Gowda M, Liu W, Wurschum T, Maurer HP, Longin FH, Ranc N, Reif JC (2012). Accuracy of genomic selection in European maize elite breeding populations. Theor Appl Genet.

[CR75] Zhu JK (2016). Abiotic stress signaling and responses in plants. Cell.

[CR76] Zhu Z, Zhang F, Hu H, Bakshi A, Robinson MR, Powell JE, Montgomery GW, Goddard ME, Wray NR, Visscher PM, Yang J (2016). Integration of summary data from GWAS and eQTL studies predicts complex trait gene targets. Nat Genet.

